# Differences in the Psychological Profiles of Elite and Non-elite Athletes

**DOI:** 10.3389/fpsyg.2021.635651

**Published:** 2021-03-18

**Authors:** Petar Mitić, Jasmina Nedeljković, Željka Bojanić, Mirjana Franceško, Ivana Milovanović, Antonino Bianco, Patrik Drid

**Affiliations:** ^1^Faculty of Sport and Physical Education, University of Niš, Niš, Serbia; ^2^Faculty of Legal and Business Studies Dr Lazar Vrkatic, Novi Sad, Serbia; ^3^Faculty of Sport and Physical Education, University of Novi Sad, Novi Sad, Serbia; ^4^Department of Psychology, Educational Science and Human Movement, Sport and Exercise Sciences Research Unit, University of Palermo, Palermo, Italy

**Keywords:** sport, self-efficacy, time perspective, emotional intelligence, achievement motivation, personality dimensions

## Abstract

One of the main goals of sport psychology is to identify those psychological factors that are relevant for sport performance as well as possibilities of their development. The aim of the study was to determine whether the set of specific psychological characteristics [generalized self-efficacy, time perspective, emotional intelligence (EI), general achievement motivation, and personality dimensions] makes the distinction between athletes based on their (non)-participation in the senior national team, that is, their belonging to the subsample of elite or non-elite athletes depending on this criterion. According to the group centroids it can be said that elite athletes are characterized by a positive high score in self-efficacy, emotionality, present fatalistic time perspective, past positive time perspective, and openness to experience. They are also characterized by low past negative time perspective, emotional competence, and future time perspective. Non-elite athletes have the opposite traits. The results have been discussed in the context of their application in the process of talent selection and development in sport as well as the development of life skills in athletes.

## Introduction

Researchers and practitioners in the field of sport psychology have always been interested in the psychological factors that affect sport performance. Some studies have shown that it is possible to predict future success in sport, based on specific psychological factors, relatively successfully even in an early stage of sports engagement (Van Yperen, 2009), and that psychological factors are crucial and should be developed from an early sports age (MacNamara et al., 2010). In this regard, the Talent Identification and Development System (TIDS) has been designed, which consists of four steps including Talent Detection (refers to those who have not been involved in sports yet), Talent Identification (early talent detection in athletes), Talent Development (providing the most favorable conditions for the achievement of assumed potentials), and Talent Selection (standard form of sport selection) (Reilly et al., 2000; Till and Baker, 2020). Talent Transfer, which implies guiding athletes toward the sports in which they can realize the maximum of their potentials, has been included later in the process (MacNamara and Collins, 2015). As emphasized by some critical publications (Johnston and Baker, 2019), the decision-making process regarding talent in sport is sometimes not so objective, which results in some talented children “dropping out”. As for the complexity and challenges related to the TIDS, Till and Baker (2020) recommended that talent development should be applied to a wider sports population (as much as possible), since this would be more ethical as well as more useful in terms of the improvement of both health and performance.

The psychological variables considered in the context of sport performance in this study included: self-efficacy, time perspective, emotional intelligence (EI), general achievement motivation, and personality dimensions (emotionality, extraversion, agreeableness, conscientiousness, openness to experience, and honesty-humility). The choice of mentioned variables does not derive from a special, coherent model, but is made based on the criteria of the possibility for development and relationships with successful functioning inside and outside the sports context. So, in addition to previous studies which have indicated their relationship with sport performance (excluding time perspectives), there were two more reasons to include these variables in the study. The first reason for selecting these variables was a possibility of their improvement (except for some of the personality dimensions), which is relevant for potential talent development programs in sport. The second reason was a definite correlation between these variables and the functioning of persons outside the context of sports. Although psychologists, during a sports career, are primarily focused on improving athletes' performances, what happens to athletes after the end of their sports careers and transition to other ones should not be neglected (Taylor and Lavallee, 2009).

According to the standard definition, EI consists of four attributes: (1) ability to perceive, assess, and express emotions quickly, (2) ability to recognize and generate the feelings that facilitate thinking, (3) ability to understand emotions and knowledge about emotions, (4) ability to manage emotions in order to improve emotional and intellectual development (Salovey and Mayer, 1990). Although everybody mostly agrees with the above definition, there is also a dilemma regarding its operationalization in measurement—whether it should be considered as an ability or a personality trait. Observing and measuring EI as an ability means to measure it as done in the case of standard tests used for measuring IQ, i.e., by the application of achievement tests which involve correct and incorrect answers (Petrides, 2011) such as MEIS (Mayer et al., 1999) and MSCEIT (Mayer et al., 2002) tests. Should it be considered as a trait, the operationalization is performed using self-assessment questionnaires and it is often called a trait of emotional self-efficacy (Petrides, 2011). There are many EI self-assessment questionnaires (e.g., EQ-i—Bar-On, 1997; SEIS—Schutte et al., 1998; TEIQue—Petrides and Furnham, 2001; ECI—Boyatzis et al., 1999; UEK-45—Takšić, 2002).

A high level of EI is associated with using psychological techniques such as self-talk, imaginary, and activation more frequently, both in training and competitions (Lane et al., 2009). Having studied running, which requires a great endurance (282 km in 6 days), Lane and Wilson (2011) found that the athletes with a higher level of emotional intelligence trait experienced pleasant emotions more often in relation to unpleasant ones. The trait of EI correlated with a number of points and a number of matches played in professional ice-hockey (Perlini and Halverson, 2006), satisfaction with sport performance (Laborde et al., 2014), and frequency and duration of practicing both individual and team sports (Laborde et al., 2017). Also, when it comes to the studies on EI as an ability, the obtained results have suggested that it is a significant predictor of sport performance in cricket (Crombie et al., 2009). To a certain extent, EI can predict life skills (Bastian et al., 2005), and it is associated with performance in the workplace as well (O'Boyle et al., 2011; Joseph et al., 2015). The studies have shown that EI can be developed under various conditions (Bagshaw, 2000; Slaski and Cartwright, 2005), including sport (Campo et al., 2016).

A time perspective means that a person is dominantly focused on thinking about their past, present, or future and it has a dynamic impact on the person's experience, motivation, opinion, and other forms of behavior, i.e., it represents a personal attitude toward time (Zimbardo and Boyd, 1999). Zimbardo and Boyd (1999, 2008) stated that the past, present, and future time perspectives may include the following operationally defined perspectives: past-negative, past-positive, present-hedonistic, present-fatalistic, future. The recent approaches to the study of time perspectives (Stolarski et al., 2018) have suggested two additional dimensions (Future-Negative and Present-Eudaimonic), as well as a dual perception of time perspectives—considered as a trait or a state.

Future-positive time perspective is in a positive correlation with physical activity in adolescents (Henson et al., 2006). Some authors (Zentsova and Leonov, 2013) found past-positive time perspective to be dominant in professional athletes, and, therefore, recommended that coaches and sport psychologists should put their efforts to change such a situation and influence the development of future time perspective in athletes. As they assumed, this would contribute to the athletes not being satisfied with already achieved results, which could help coaches to set goals more adequately. Some studies have indicated that time perspectives can be changed by interventions (Oyanadel et al., 2014). Balanced Time Perspective can be developed and is important for adaptations in human functioning (Stolarski et al., 2020). Stolarski et al. (2019) provided a complex model of possible effects of time perspectives on sport performance, which needs an empirical validation.

Bandura (1999) defined self-efficacy as an individual's assessment of his or her own capacities to organize and execute specific actions necessary to achieve desired goals. Self-efficacy reflects a person's confidence in their own ability to exert complete control over the outcomes of the previously set goals, in spite of events, difficulties, or obstacles that could interfere with goal achievement (Bandura, 1986). Self-efficacy has been recognized as a significant factor in the context of sport (Feltz et al., 2008). Some authors have considered self-efficacy as an important attribute relevant for understanding variations in sport competition anxiety (Wittig et al., 1987). A high level of perceived self-efficacy reduces the chances of an athlete participating in self-handicapping behavior, i.e., finding a reason for poor performance in advance (Kuczka and Treasure, 2005), and it is, therefore, recommended that coaches work on enhancing self-efficacy. The perceived self-adequacy related to physical activity can affect running results in children (Cairney et al., 2008). General self-efficacy is a significant factor which can moderate an impact and interpretation of the relationship between personality traits and perceived stress (Ebstrup et al., 2011). The meta-analysis of 45 studies has found that the average correlation between self-efficacy and sport performance is 0.38 (Moritz et al., 2000). Most studies apply specific scales for the assessment of self-efficacy (Feltz and Lirgg, 2001). Self-efficacy can be developed in athletes successfully through various interventions (Zagórska and Guszkowska, 2014). Generalized self-efficacy represents a protective factor in stressful life transitions (Jerusalem and Mittag, 1995) and it is one of the best predictors of performance in the workplace (Judge and Bono, 2001).

Athletes' motivation is one of the most important topics considered in sport psychology (Aktop and Erman, 2006) and achievement motivation is one of the most researched fields from the aspect of various theoretical approaches (Ong, 2019). Achievement motives in athletes are different in regard to gender, type of sport, and level of competition (Van de Pol and Kavussanu, 2012; Ong, 2019). The research suggests that a proper development of achievement motivation can prevent a premature drop-out in sport (Gardner et al., 2017), as well as that achievement motivation is associated with moral reasoning in athletes (Tod and Hodge, 2001). In this paper, we have taken the stand stating that a real value of the achievement motivation measurement is not reflected in predicting success in competitions but in predicting long-term types of motivation (Cox, 1998). Achievement motivation is in correlation with entrepreneurial career and performance (Collins et al., 2004).

The five-factor model of personality (McCrae and John, 1992; McCrae and Costa, 2008), which includes the following dimensions: extraversion, neuroticism, openness, agreeableness, and conscientiousness, represents the dominant model applied in personality research. The HEXACO model of basic personality structure (Lee and Ashton, 2008) was created by testing the “Big five” model when, in further lexical studies, it turned out necessary to examine the six-factor structure or the structure of latent dimensions underlying the personality descriptors in different languages. The sixth factor of personality has been confirmed in the majority of operationalizations of this model and it is called “honesty/humility” (Ashton et al., 2004). Numerous studies have confirmed that specific dimensions of personality are differently expressed in athletes of different levels of competition (e.g., Garland and Barry, 1990; Allen et al., 2011). Also, it seems absolutely justified to put forward the question concerning how participation in sports and competitions can affect the development of personality (Allen et al., 2013), regardless of its strong genetic basis. Personality development is significant since, in a non-sport context, personality traits are associated with a number of important psychological and behavioral factors such as coping with stress (Connor-Smith and Flachsbart, 2007), successful task performance (Oh et al., 2011), unhealthy life habits such as smoking and alcohol abuse (Malouff et al., 2006, 2007).

The aim of this study was to determine whether the set of specific psychological characteristics (generalized self-efficacy, time perspective, EI, general achievement motivation, and personality dimensions) makes the distinction between athletes based on their (non)-participation in the senior national team, that is, their belonging to the subsample of elite or non-elite athletes depending on this criterion.

## Materials and Methods

### Participants

The sample of this study included 230 athletes (22.35 ± 4.16 years) from the Republic of Serbia (140 males and 90 females). The criterion according to which the sample was divided was their participation in the Serbian senior national teams. The national team members (who all have significant experience at an international level as well) included 94 athletes from the sample, while the remaining part of the sample (136 athletes) included the athletes participating in national competitions, training more than five times a week, but have never been invited to join a senior national team and have no international experience. The inclusion criteria in the research also was that athletes train for more than 7 years. The sample of the athletes who are not the members of national teams was proportionate to the sample of the athletes who appear in the national teams, according to gender and type of sport. Such a division was made to clearly distinguish between the elite athletes and the athletes who cannot be classified as the elite ones. The entire Serbian senior national teams (male basketball national team, male volleyball national team, female volleyball national team, female football national team), as well as individual members of the male and female judo national teams and members of water polo and handball national teams, participated in this study. All participants were completing a paper and pencil questionnaires.

### Instruments

The *Zimbardo Time Perspective Inventory* (Zimbardo and Boyd, 1999), or more precisely the Serbian version of this instrument (ZTPI, Kostić and Nedeljković, 2013) comprising 52 items was used to examine time perspectives. The dimensions are the same as in the original questionnaire: *past-negative (8 items), present-hedonistic (16 items), future (11 items), past-positive (13 items)*, and *present-fatalistic (4 items)*. The reliability of the whole questionnaire obtained in this study by calculating the Cronbach alpha is 0.868.

The instrument used to assess self-efficacy was the *General Self*-*Efficacy Scale* (GSE, Schwarzer and Jerusalem, 1995) which consists of 10 items, and respondents provide answers showing the extent to which the given statements are true in relation to themselves, using a five-point Likert-type scale. The reliability of the questionnaire obtained in this study by calculating the Cronbach alpha is 0.827.

The instrument used to determine a level of achievement motivation was *MOP2002* (Franceško et al., 2002) which consists of 55 items formulated as statements, and the respondents assessed a degree of agreement using a five-point scale. This scale includes the following four factors of the first order: Persistence in achieving goals (15 items), Competing with others (19 items), Goal achievement as a source of satisfaction (13 items), and Orientation toward planning (8 items). Since the specified factors were not independent and significantly correlated with the overall score, only the total score was used in the study. The reliability of the questionnaire obtained in this study by calculating the Cronbach alpha is 0.905.

The instrument used to determine personality traits was *HEXACO-PI-R* (Lee and Ashton, 2018) which consists of 100 items in total. The dimensions represented were as follows: *Honesty, Emotionality, Extraversion, Agreeableness, Conscientiousness*, and *Openness*. The Serbian version showed good metric characteristics (Mededović et al., 2019). The reliability of the questionnaire obtained in this study by calculating the Cronbach alpha is 0.866.

*The emotional competence questionnaire* (Takšić, 2002) consists of 45 statements where respondents give their answers by choosing one of the numbers given on a five-point scale. The answers represent the respondents' assessment of the development of their own abilities regarding emotional competence. In addition to the overall score, the scores using the subscales of *Ability to perceive and understand emotions, Ability to express and identify emotions*, and *Ability to manage and regulate emotions* were also obtained. Only the overall score was used in the study. The reliability of the questionnaire obtained in this study by calculating the Cronbach alpha is 0.909.

### Data Analysis

In the data analysis, descriptive measures, mean differences, and discriminant analysis were used.

## Results

The basic research question was whether a set of psychological factors including time perspectives, self-efficacy, basic personality traits, general achievement motive, and EI made a distinction between the athletes according to their participation in the national teams. Since univariate tests do not take into account the relationship among variables, a discriminant function analysis was also performed on the 14 variables. The values of group centroids (mean discriminant scores for each group) for the national team members and the non-member athletes were 0.599 and −0.414, respectively. The discriminant function obtained for the 14 factors, Wilks λ = 0.800, Chi-square (14) = 49.312, *p* < 0.001. Refer to Table 1 for the entry order of the 14 variables and their corresponding standardized discriminant function coefficients.

**Table 1:** Means, standard deviations, *t*-tests, standardized discriminant function coefficients, and correlations between discriminant scores and variable raw scores for national team members and non-members.

	**National team**	**Mean**	***SD***	***F*_**(1, 228)**_**	**Stand. Can. Disc. Fun. Coeff**.	**Corr. Disc. Var. Raw**
Past negative TP	Yes	2.04	0.721	7.699^**^	−0.693	−0.368
	No	2.33	0.847			
Present hedonistic TP	Yes	3.58	0.566	2.661	0.022	0.216
	No	3.47	0.443			
Past positive TP	Yes	3.22	0.692	0.008	0.132	0.012
	No	3.21	0.621			
Future TP	Yes	3.54	0.515	1.043	−0.250	0.135
	No	3.47	0.527			
Present fatalistic TP	Yes	2.38	0.715	0.000	0.247	0.001
	No	2.38	0.833			
Self-efficacy	Yes	34.78	3.558	28.698^**^	0.981	0.710
	No	31.99	4.101			
Honesty-Humility	Yes	53.41	9.407	0.140	−0.089	0.050
	No	52.96	8.707			
Emotionality	Yes	49.14	7.386	2.452	0.594	0.207
	No	47.69	6.615			
Extraversion	Yes	59.61	7.569	3.370	0.036	0.243
	No	57.67	8.089			
Agreeableness	Yes	47.42	7.591	0.522	0.062	−0.096
	No	48.08	6.272			
Conscientiousness	Yes	59.13	8.821	2.664	−0.031	0.216
	No	57.33	7.841			
Openness to experience	Yes	54.53	9.753	2.401	0.130	0.205
	No	52.65	8.501			
General motive of achievement	Yes	212.48	20.964	3.871^*^	0.055	0.261
	No	207.29	18.755			
Emotional competence	Yes	172.97	18.816	2.512	−0.380	0.210
	No	169.19	17.031			

* *p < 0.05*;

** *p < 0.01*.

The obtained canonical discriminant function is: Discriminant score = 0.981 ^*^ self-efficacy + 0.594 ^*^ emotionality + 0.247 ^*^ present fatalistic time perspective + 0.132 ^*^ past positive time perspective + 0.130 ^*^ openness to experience – 0.693 ^*^ past negative time perspective – 0.380 ^*^ emotional competence – 0.250 ^*^ future time perspective.

According to the group centroids it can be said that the members of the national teams are characterized by a positive high score in self-efficacy, emotionality, present fatalistic time perspective, past positive time perspective, and openness to experience. They are also characterized by low past negative time perspective, emotional competence, and future time perspective.

## Discussion

The obtained results have shown that self-efficacy is the most important factor which made a distinction between the athletes who are members of the national teams and those who are non-members, i.e., successful and less successful athletes. General self-efficacy is one's confidence in the ability to overcome demanding situations and one's belief that success depends on our own actions (Schwarzer and Jerusalem, 1995). Taking into account that the sources of self-efficacy in sport (past performance accomplishments, social/verbal persuasion, vicarious experience/modeling, and interpretation of physical/emotional states) are the same as in other fields of human functioning (Samson and Solmon, 2011), it is easy to assume why such big differences occurred in the perceived self-efficacy—more successful athletes had a greater number of and more significant results, therefore, they were certainly more convinced of their own capabilities related to sport achievements via the media and their sports environment. One of the methods to enhance self-efficacy in the sport context is to practice self-talk (Hatzigeorgiadis et al., 2008). However, the obtained results gain in significance, taking into account that generalized self-efficacy, and not the sport-specific one, was examined in this study, which was not the case in most studies which dealt with the relationship between self-efficacy and sport performance (Feltz and Lirgg, 2001). This practically means that more successful athletes are confident about their abilities not only when it comes to sports venues but also in general. Since a high level of self-efficacy is associated with life transitions and work-related performance, it may be assumed that they are more prepared for a more successful career transition following the termination of their active sport participation. Thus, the findings obtained in this study suggest that the development of general self-efficacy should be included in the talent development programs in sport as well as that sport performance would be thereby improved and life skills developed.

Personality dimensions defined by the HEXACO Model (Ashton et al., 2014) are another psychological factor which distinguishes between the elite and non-elite athletes, as suggested by the results of this study. More successful athletes are characterized by higher levels of emotionality and openness to experience. Emotionality comprises subordinating aspects: Fearfulness, Anxiety, Dependence, and Sentimentality, and high scores on this scale are achieved by the persons who fear of physical dangers of injuries, they feel anxiety as a response to life stresses and the need for emotional support by other people, have empathy and sentimentality for others (Ashton et al., 2014). Logically, body and its readiness to perform is a basic precondition of success in sport and elite athletes are aware of the fact that injuries make the desired sport results more distant so they feel fear of injuries. High scores related to the need for emotional support by other people, empathy and sentimentality may be described by the fact that a vast majority of the respondents included in this study are engaged in team sports and mutual support and understanding are often crucial for successful team play. Both elite and non-elite athletes show a higher level of anxiety in relation to general population (Matsumoto et al., 2000), and a great percentage of athletes achieve the best results in the state of intensified anxiety (Hanin, 2007). Higher scores related to the dimension of openness to experience may indicate the creativity in performance and an athlete's responsiveness to coaches' various tactical and training suggestions. It should be noted that these interpretations of the obtained findings are speculative to a great extent, since the facets comprising the domains were excluded from the statistical processing in this study. In regard to the role of global personality dimensions in sport, the seemingly reasonable perspective obtained by the analysis of the so-far research should also be noted, stating that personality dimensions mediate the relationships between the factors which are specifically relevant for sport performance but do not predict success directly (Allen et al., 2013).

By examining the differences between the members of national teams (elite athletes) and those who are non-members (non-elite athletes) in regard to time perspectives, it has been found that the elite athletes are characterized by high present fatalistic and past positive time perspectives, and past negative and future time perspectives are less expressed in these athletes. Unfortunately, the number of studies that investigate time perspectives in a sports context is neglectable, so the discussion can be based on logical analysis and assumed models of the connection between playing sports and the perception of time perspectives. It was expected that the time perspectives of the elite and non-elite athletes were different since they arise out of personal and social experience (Zimbardo and Boyd, 2008), and their engagement in sports is certainly significant experience for both groups of the athletes examined. The assumed model of the effects of time perspectives on sports engagement (Stolarski et al., 2019) suggested that past positive time perspective would have positive effects, either directly or indirectly, and past negative time perspective would have adverse effects on sport performance. The results obtained in this study have supported this assumption. However, the results which are inconsistent with the assumed model were also obtained in this research. Present fatalistic time perspective has shown to be more present in the elite athletes and it was expected to have negative effects on a motivation-engagement-performance chain (Stolarski et al., 2019). The obtained data is unexpected when we take into account that people with fatalistic understanding of the present have, amongst other things, reduced personal efficiency, external locus of control and planning, which should be accompanied by effort and work. It is very difficult to connect such a description with elite athletes since they go through various cycles of carefully planned preparations during which clearly determined goals are realized. It may be possible to assume that elite athletes are satisfied with what they have achieved and do not make plans, while non-elite athletes are convinced that they can still significantly increase their performance through training. One of explanations for expression of the fatalistic present in top athletes can be found in the lack of autonomy during sports development. Lack of autonomy refers to the exclusion (although sometimes with the best intentions) of athletes from decision-making process and is associated with occurrence of overtraining in young athletes (Winsley and Matos, 2011). A lower future time perspective of the elite athletes was most difficult to explain since its significant positive impact on sport results was assumed, especially through motivation (Zentsova and Leonov, 2013; Stolarski et al., 2019). Nevertheless, a real effect of future time perspective in a sport context can be considered in different ways. For example, Stolarski et al. (2019) stated that a high future positive time perspective could be proven a factor which would prevent young athletes' drop-out, and present fatalistic time perspective would have opposite effects. This may be true given the findings of the research indicating a dominant future perspective in less successful athletes and a higher present fatalistic time perspective found in more successful athletes. Both groups of athletes remained involved in sports and the reasons for their staying in sports may be found in the future time perspective of those belonging to the group of less successful athletes—they plan and set goals and put efforts in their realization, therefore, they can delay current pleasures in anticipation of achieving a greater satisfaction in future. The reason for a long-term participation in sports in elite athletes can be probably found in their realistic sports achievements which are high in both the present and the past. Regardless of the slightly unexpected results, we hope that this study, being among the first ones of its kind, would serve as the background for further, more complex, empirical research on the theory of time perspectives in the field of competitive sports.

The results of the study have shown that the presence of a general achievement motive did not make a difference in the athletes' psychological profiles in regard to performance, and the finding stating that emotional competence (emotional intelligence trait) was more expressed in the less successful athletes was surprising, since it is contrary to the results of some previous studies (Perlini and Halverson, 2006; Laborde et al., 2014).

The aim of the study was to determine whether the set of specific psychological characteristics (generalized self-efficacy, time perspective, EI, general achievement motivation, and personality dimensions) makes the distinction between athletes based on their (non)-participation in the senior national team, that is, their belonging to the subsample of elite or non-elite athletes depending on this criterion. The study was conducted on a highly selected sample of the elite athletes (most of whom have a medal won at the Olympics or World or European Championships) and a parallel sample of the less successful athletes. The study was conducted by the application of the questionnaires which were used in the previous studies and demonstrated excellent metric characteristics in the culture the examined athletes belong to. The results have shown that the elite athletes, compared to those who are less successful, have high scores in self-efficacy, emotionality, present fatalistic time perspective, past positive time perspective, and openness to experience. They are also characterized by low past negative time perspective, emotional competence, and future time perspective.

## Limitations and Future Lines of Research

There are also some limitations regarding the results of this study. The first limitation is related to the fact that the study was conducted in one culture which relativizes the generalization and extrapolation of the obtained findings. Another limitation refers to heterogeneity of the sample in terms of gender, type of sport, and not controlling the length of sports experience (it was only important that they train for more than 7 years). The division into sub-samples obtained by crossing criteria of sex and sports performance would lead to far too small sub-samples for carrying out valid conclusions based on statistical analysis. However, we believe that such heterogeneous samples also served to establish a general distinction between elite and non-elite athletes, and laid foundations for further research on larger samples. This is also justified by the fact that data obtained in this study actually aim to suggest an improvement in the long-term development of young athletes regardless of their gender, although it is certain that gender differences would contribute to clearer recommendations for sports practitioners. The third limitation could be associated with the application of instruments which are not sport-specific, but intended for general population. Regardless of the fact that the application of sport-specific instruments would (probably) demonstrate clearer differences between the subsamples there are two reasons for opting for this instrument. The first being a lack of sport-specific questionnaires developed for all the studied variables (personality dimensions, time perspectives). Secondly, we intended to draw attention to the fact that specific psychological traits can be developed with double benefit—to improve sport performance but also to develop life skills. In future studies, the focus should be on researching time perspectives in sports, i.e., their connection with motivational processes, stress, and overtraining, especially in young athletes. We believe that the talent development programs in young athletes have to take into account the humanistic approach to work, regardless of the extreme competitiveness and, sometimes, even cruelty of today's sport. Talents should definitely be developed, however, during this process, the care should be taken of the children who do not manage to become top athletes for various reasons, as well as of athletes after the termination of their active sports careers.

## Data Availability Statement

The raw data supporting the conclusions of this article will be made available by the authors, without undue reservation.

## Ethics Statement

The studies involving human participants were reviewed and approved by Institutional Review Committee of the University of Novi Sad (Ref. No. 46-06-02/2020-1). The patients/participants provided their written informed consent to participate in this study.

## Author Contributions

PM, JN, ŽB, and MF wrote the article. PM, JN, ŽB, MF, IM, AB, and PD designed the study, analyzed the data, discussed the results, and reviewed and approved the article. All authors contributed to the article and approved the submitted version.

## Conflict of Interest

The authors declare that the research was conducted in the absence of any commercial or financial relationships that could be construed as a potential conflict of interest. The reviewer KB declared a past collaboration with several of the authors, IM, AB, and PD.

## References

[B1] Aktop A. Erman K. A. (2006). Relationship between achievement motivation, trait anxiety and self-esteem. Biol. Sport. 23, 127–141.

[B2] Allen M. S. Greenlees I. Jones M. V. (2011). An investigation of the five-factor model of personality and coping behaviour in sport. J. Sports Sci. 29, 841–850. 10.1080/02640414.2011.565064 21500081

[B3] Allen M. S. Greenlees I. Jones M. V. (2013). Personality in sport: a comprehensive review. Int. Rev. Sport Exerc. Psychol. 6, 184–208. 10.1080/1750984X.2013.769614

[B4] Ashton M. C. Lee K. De Vries R. E. (2014). The HEXACO honesty-humility, agreeableness, and emotionality factors: a review of research and theory. Pers. Soc. Psychol. Rev. 18, 139–152. 10.1177/1088868314523838 24577101

[B5] Ashton M. C. Lee K. Perugini M. Szarota P. de Vries R. E. Di Blas L.. (2004). A six-factor structure of personality-descriptive adjectives: solutions from psycholexical studies in seven languages. J. Pers. Soc. Psychol. 86, 356–366. 10.1037/0022-3514.86.2.356 14769090

[B6] Bagshaw M. (2000). Emotional intelligence–training people to be affective so they can be effective. Ind. Commer. Train 32, 61–65. 10.1108/00197850010320699

[B7] Bandura A. (1986). The explanatory and predictive scope of self-efficacy theory. J. Soc. Clin. Psychol. 4, 359–373. 10.1521/jscp.1986.4.3.359

[B8] Bandura A. (1999). A social cognitive theory of personality, in Handbook of Personality, 2nd Edn., eds Pervin L. John O. (New York, NY: Guilford Publications), 154–196.

[B9] Bar-On R. (1997). The Emotional Intelligence Inventory (EQ–i): Technical Manual. Toronto, ON: Multi-Health Systems.

[B10] Bastian V. A. Burns N. R. Nettelbeck T. (2005). Emotional intelligence predicts life skills, but not as well as personality and cognitive abilities. Pers. Individ. Differ. 39, 1135–1145. 10.1016/j.paid.2005.04.006

[B11] Boyatzis R. E. Goleman D. Hay M. (1999). Emotional Competence Inventory. Boston, MA: HayGroup.

[B12] Cairney J. Hay J. A. Faught B. E. Leger L. U. C. Mathers B. (2008). Generalized self-efficacy and performance on the 20-metre shuttle run in children. Am. J. Hum. Biol. 20, 132–138. 10.1002/ajhb.20690 17990324

[B13] Campo M. Laborde S. Mosley E. (2016). Emotional intelligence training in team sports. J. Individ. Differ. 37, 152–158. 10.1027/1614-0001/a000201

[B14] Collins C. J. Hanges P. J. Locke E. A. (2004). The relationship of achievement motivation to entrepreneurial behavior: a meta-analysis. Hum. Perform. 17, 95–117. 10.1207/S15327043HUP1701_5

[B15] Connor-Smith J. K. Flachsbart C. (2007). Relations between personality and coping: a meta-analysis. J. Pers. Soc. Psychol. 93:1080. 10.1037/0022-3514.93.6.1080 18072856

[B16] Cox R. H. (1998). Sport Psychology: Concepts and Applications. Boston, MA: McGraw-Hill.

[B17] Crombie D. Lombard C. Noakes T. (2009). Emotional intelligence scores predict team sports performance in a National cricket competition. Int. J. Sports. Sci. Coach. 4, 209–224. 10.1260/174795409788549544

[B18] Ebstrup J. F. Eplov L. F. Pisinger C. Jørgensen T. (2011). Association between the Five Factor personality traits and perceived stress: is the effect mediated by general self-efficacy? Anxiety. Stress. Coping. 24, 407–419. 10.1080/10615806.2010.540012 21213153

[B19] Feltz D. L. Lirgg C. D. (2001). Self-efficacy beliefs of athletes, teams, and coaches, in Handbook of Sport Psychology, 2nd Edn., eds Singer R. N. Hausenblas H. A. Janelle C. (New York, NY: John Wiley and Sons), 340–361.

[B20] Feltz D. L. Short S. E. Sullivan P. J. (2008). Self-Efficacy in Sport. Champaign, IL: Human Kinetics.

[B21] Franceško M. Mihić V. Bala G. (2002). Struktura motiva postignuća merena skalom MOP 2002 [Structure of achievement motive measured by the MOP 2002 scale], in Ličnost u Višekulturnom Društvu: Organizacijska Multikulturalnost i Evropski Identitet [Personality in a Multicultural Society: Organizational Multiculturalism and European Identity], eds Cukić B. Franceško M. (Novi Sad: Filozofski fakultet), 134–143.

[B22] Gardner L. A. Vella S. A. Magee C. A. (2017). Continued participation in youth sports: the role of achievement motivation. J. Appl. Sport. Psychol. 29, 17–31. 10.1080/10413200.2016.1173744

[B23] Garland D. J. Barry J. R. (1990). Personality and leader behaviors in collegiate football: a multidimensional approach to performance. J. Res. Pers. 24, 355–370. 10.1016/0092-6566(90)90026-3

[B24] Hanin Y. L. (2007). Emotions and athletic performance: individual zones of optimal functioning model, in Essential Readings in Sport and Exercise Psychology, eds Smith D. Bar-Eli M. (Champaign, IL: Human Kinetics), 55–73.

[B25] Hatzigeorgiadis A. Zourbanos N. Goltsios C. Theodorakis Y. (2008). Investigating the functions of self-talk: the effects of motivational self-talk on self-efficacy and performance in young tennis players. Sport. Psychol. 22, 458–471. 10.1123/tsp.22.4.458

[B26] Henson J. M. Carey M. P. Carey K. B. Maisto S. A. (2006). Associations among health behaviors and time perspective in young adults: model testing with boot-strapping replication. J. Behav. Med. 29, 127–137. 10.1007/s10865-005-9027-2 16421652 PMC2435266

[B27] Jerusalem M. Mittag W. (1995). Self-efficacy in stressful life transitions, in Self-Efficacy in Changing Societies, ed A. Bandura (Cambridge, UK: Cambridge University Press), 177–201. 10.1017/CBO9780511527692.008

[B28] Johnston K. Baker J. (2019). Waste reduction strategies: factors affecting talent wastage and the efficacy of talent selection in sport. Front. Psychol. 10:295. 10.3389/fpsyg.2019.02925 31998188 PMC6967295

[B29] Joseph D. L. Jin J. Newman D. A. O'Boyle E. H. (2015). Why does self-reported emotional intelligence predict job performance? A meta-analytic investigation of mixed EI. J. Appl. Psychol. 100, 298–342. 10.1037/a0037681 25243996

[B30] Judge T. A. Bono J. E. (2001). Relationship of core self-evaluations traits—self-esteem, generalized self-efficacy, locus of control, and emotional stability—with job satisfaction and job performance: a meta-analysis. J. Appl. Psychol. 86, 80–92. 10.1037/0021-9010.86.1.80 11302235

[B31] Kostić A. Nedeljković J. (2013). Studije Vremenskih Perspektiva u Srbiji. [Time Perspectives Studies in Serbia]. Niš: Punta.

[B32] Kuczka K. K. Treasure D. C. (2005). Self-handicapping in competitive sport: influence of the motivational climate, self-efficacy, and perceived importance. Psychol. Sport. Exerc. 6, 539–550. 10.1016/j.psychsport.2004.03.007

[B33] Laborde S. Dosseville F. Guillén F. Chávez E. (2014). Validity of the trait emotional intelligence questionnaire in sports and its links with performance satisfaction. Psychol. Sport. Exerc. 15, 481–490. 10.1016/j.psychsport.2014.05.001

[B34] Laborde S. Guillén F. Watson M. (2017). Trait emotional intelligence questionnaire full-form and short-form versions: links with sport participation frequency and duration and type of sport practiced. Pers. Individ. Differ. 108, 5–9. 10.1016/j.paid.2016.11.061

[B35] Lane A. M. Thelwell R. C. Lowther J. Devonport T. J. (2009). Emotional intelligence and psychological skills use among athletes. Soc. Behav. Pers. 37, 195–201. 10.2224/sbp.2009.37.2.195

[B36] Lane A. M. Wilson M. (2011). Emotions and trait emotional intelligence among ultra-endurance runners, J. Sci. Med. Sport. 14, 358–362. 10.1016/j.jsams.2011.03.001 21440500

[B37] Lee K. Ashton M. C. (2008). The HEXACO personality factors in the indigenous personality lexicons of English and 11 other languages. J. Pers. 76, 1002–1054. 10.1111/j.1467-6494.2008.00512.x 18665898

[B38] Lee K. Ashton M. C. (2018). Psychometric properties of the HEXACO-100. Assessment 25, 543–556. 10.1177/1073191116659134 27411678

[B39] MacNamara Á. Button A. Collins D. (2010). The role of psychological characteristics in facilitating the pathway to elite performance part 1: identifying mental skills and behaviors. Sport. Psychol. 24, 52–73. 10.1123/tsp.24.1.52

[B40] MacNamara Á. Collins D. (2015). Profiling, exploiting, and countering psychological characteristics in talent identification and development. Sport. Psychol. 29, 73–81. 10.1123/tsp.2014-0021

[B41] Malouff J. M. Thorsteinsson E. B. Rooke S. E. Schutte N. S. (2007). Alcohol involvement and the five-factor model of personality: a meta-analysis. J. Drug. Educ. 37, 277–294. 10.2190/DE.37.3.d 18047183

[B42] Malouff J. M. Thorsteinsson E. B. Schutte N. S. (2006). The five-factor model of personality and smoking: a meta-analysis. J. Drug. Educ. 36, 47–58. 10.2190/9EP8-17P8-EKG7-66AD 16981639

[B43] Matsumoto D. Takeuchi M. Nakajima T. Iida E. (2000). Competition anxiety, self-confidence, personality and competition performance of American elite and non-elite judo athletes. Res. J. Budo. 32, 12–21. 10.11214/budo1968.32.3_12

[B44] Mayer J. D. Caruso D. R. Salovey P. (1999). Emotional intelligence meets traditional standards for an intelligence. Intelligence 27, 267–298. 10.1016/S0160-2896(99)00016-112934682

[B45] Mayer J. D. Salovey P. Caruso D. R. (2002). The Mayer-Salovey-Caruso Emotional Intelligence Test (MSCEIT): User's Manual. Toronto, ON: Multi–Health Systems.

[B46] McCrae R. R. Costa P. T. (2008). The five-factor theory of personality, in Handbook of Personality: Theory and Research, 3rd Edn., eds John O. P. Robins R. W. Pervin L. A. (New York, NY: Guilford Press), 159–181.

[B47] McCrae R. R. John O. P. (1992). An introduction to the five-factor model and its applications. J. Pers. 60, 175–215. 10.1111/j.1467-6494.1992.tb00970.x 1635039

[B48] Mededović J. Colović P. Dinić B. M. Smederevac S. (2019). The HEXACO personality inventory: validation and psychometric properties in the Serbian language. J. Pers. Assess. 101, 25–31. 10.1080/00223891.2017.1370426 28980831

[B49] Moritz S. E. Feltz D. L. Fahrbach K. R. Mack D. E. (2000). The relation of self-efficacy measures to sport performance: a meta-analytic review. Res. Q. Exerc. Sport. 71, 280–294. 10.1080/02701367.2000.10608908 10999265

[B50] O'Boyle E. H. Jr. Humphrey R. H. Pollack J. M. Hawver T. H. Story P. A. (2011). The relation between emotional intelligence and job performance: a meta-analysis. J. Organ. Behav. 32, 788–818. 10.1002/job.714

[B51] Oh I. S. Wang G. Mount M. K. (2011). Validity of observer ratings of the five-factor model of personality traits: a meta-analysis. J. Appl. Psychol. 96, 762–773. 10.1037/a0021832 21142341

[B52] Ong N. C. (2019). Assessing objective achievement motivation in elite athletes: a comparison according to gender, sport type, and competitive level. Int. J. Sport. Exerc. Psychol. 17, 397–409. 10.1080/1612197X.2017.1349822

[B53] Oyanadel C. Buela-Casal G. Araya T. Olivares C. Vega H. (2014). Percepción del tiempo: resultados de una intervención grupal breve para el cambio del perfil temporal [Time perception: results of a brief group intervention to change time perspective profiles]. Suma. Psicol. 21, 1–7. 10.1016/S0121-4381(14)70001-3

[B54] Perlini A. H. Halverson T. R. (2006). Emotional intelligence in the National Hockey League. Can. J. Behav. Sci. 38, 109–119. 10.1037/cjbs2006001

[B55] Petrides K. V. (2011). Ability and trait emotional intelligence, in The Wiley-Blackwell Handbook of Individual Differences, eds Chamorro-Premuzic T. von Stumm S. Furnham A. (Cambridge, UK: Blackwell Publishing Ltd.), 656–678. 10.1002/9781444343120.ch25

[B56] Petrides K. V. Furnham A. (2001). Trait emotional intelligence: psychometric investigation with reference to established trait taxonomies. Eur. J. Pers. 15, 425–448. 10.1002/per.416

[B57] Reilly T. Williams A. M. Nevill A. Franks A. (2000). A multidisciplinary approach to talent identification in soccer. J. Sports. Sci. 18, 695–702. 10.1080/02640410050120078 11043895

[B58] Salovey P. Mayer J. D. (1990). Emotional intelligence. Imagin. Cogn. Pers. 9, 185–211. 10.2190/DUGG-P24E-52WK-6CDG

[B59] Samson A. Solmon M. (2011). Examining the sources of self-efficacy for physical activity within the sport and exercise domains. Int. Rev. Sport. Exerc. Psychol. 4, 70–89. 10.1080/1750984X.2011.564643

[B60] Schutte N. S. Malouff J. M. Hall L. E. Haggerty D. J. Cooper J. T. Golden C. J.. (1998). Development and validation of a measure of emotional intelligence. Pers. Individ. Differ. 25, 167–177. 10.1016/S0191-8869(98)00001-426176668

[B61] Schwarzer R. Jerusalem M. (1995). Generalized self-efficacy scale, in Measures in Health Psychology: A User's Portfolio. Causal and Control Beliefs, eds Weinman J. Wright S. Johnston M. (Windsor, UK: NFER–NELSON), 35–37.

[B62] Slaski M. Cartwright S. (2005). Emotional intelligence training and its implications for stress, health and performance. Stress. Health. 19, 233–239. 10.1002/smi.979

[B63] Stolarski M. Fieulaine N. Zimbardo P. G. (2018). Putting time in a wider perspective: the past, the present, and the future of time perspective theory, in The SAGE Handbook of Personality and Individual Differences, eds Zeigler-Hill V. Shackelford T. K. (Thousand Oakes, CA: SAGE Publishing), 592–628.

[B64] Stolarski M. Waleriańczyk W. Pruszczak D. (2019). Introducing temporal theory to the field of sport psychology: toward a conceptual model of time perspectives in athletes' functioning. Front. Psychol. 9:2772. 10.3389/fpsyg.2018.02772 30687202 PMC6336711

[B65] Stolarski M. Zajenkowski M. Jankowski K. S. Szymaniak K. (2020). Deviation from the balanced time perspective: a systematic review of empirical relationships with psychological variables. Pers. Individ. Differ. 156:109772. 10.1016/j.paid.2019.109772

[B66] Takšić V. (2002). Upitnici emocionalne kompetentnosti (inteligencije). [Questionnaires for emotional competence (intelligence)], (Croatian), in Zbirka Psihologijskih Mjernih Instrumenata [Collection of Psychological Measurement Instruments], eds Lackovic-Grgin K. Penezic Z. (Hrvatska: Filozofski fakultet u Zadru), 27–45.

[B67] Taylor J. Lavallee D. (2009). Career transition among athletes: is there life after sports?, in Applied Sport Psychology: Personal Growth to Peak Performance, 6th Edn., ed J. M. Williams (Columbus, OH: McGraw Hill), 542–562.

[B68] Till K. Baker J. (2020). Challenges and [possible] solutions to optimizing talent identification and development in sport. Front. Psychol. 11:664 10.3389/fpsyg.2020.00664 32351427 PMC7174680

[B69] Tod D. Hodge K. (2001). Moral reasoning and achievement motivation in sport: a qualitative inquiry. J. Sport. Behav. 24, 307–326.

[B70] Van de Pol P. K. Kavussanu M. (2012). Achievement motivation across training and competition in individual and team sports. Sport. Exerc. Perform. Psychol. 1, 91–105. 10.1037/a0025967

[B71] Van Yperen N. W. (2009). Why some make it and others do not: identifying psychological factors that predict career success in professional adult soccer. Sport. Psychol. 23, 317–329. 10.1123/tsp.23.3.317

[B72] Winsley R. Matos N. (2011). Overtraining and elite young athletes. Med. Sport Sci. 56, 97–105. 10.1159/000320636 21178369

[B73] Wittig A. F. Duncan S. L. Schurr K. T. (1987). The relationship of gender, gender-role endorsement and perceived physical self-efficacy to sport competition anxiety. J. Sport. Behav. 10, 192–199.

[B74] Zagórska A. Guszkowska M. (2014). A program to support self-efficacy among athletes. Scand. J. Med. Sci. Sports. 24, e121–e128. 10.1111/sms.12125 24118561

[B75] Zentsova N. I. Leonov S. V. (2013). Comparative characteristics of time perspective of professional athletes and drug addicted people. Proc. Soc. Behav. Sci. 78, 340–344. 10.1016/j.sbspro.2013.04.307

[B76] Zimbardo P. G. Boyd J. N. (1999). Putting time in perspective: a valid, reliable individual-differences metric. J. Pers. Soc. Psychol. 77, 1271–1288. 10.1037/0022-3514.77.6.1271

[B77] Zimbardo P. G. Boyd J. N. (2008). The Time Paradox. New York, NY: Free Press.

